# Superficial Venous Thrombosis in Non-Varicose Veins: A Narrative Review

**DOI:** 10.3390/jcm15031082

**Published:** 2026-01-29

**Authors:** Marco Mangiafico, Francesco Lorenzo Di Pino, Luca Costanzo

**Affiliations:** 1 Unit of Internal Medicine, Policlinico “G. Rodolico-San Marco” University Hospital, University of Catania, 95123 Catania, Italy; francesco.dipino@icloud.com; 2 Unit of Angiology, Cardio-Thoraco-Vascular Department, Policlinico “G. Rodolico-San Marco” University Hospital, University of Catania, 95123 Catania, Italy; lucacost84@gmail.com

**Keywords:** superficial venous thrombosis, venous thromboembolism, thrombosis, varicose vein, non-varicose vein, chronic venous insufficiency, cancer associated thrombosis, gender superficial venous thrombosis

## Abstract

**Background:** Superficial venous thrombosis (SVT) is an inflammatory and thrombotic disorder affecting superficial veins. While varicose veins (VVs) are the primary risk factor, SVT occurring in non-varicose veins (NVVs) is a critical clinical finding, often acting as a sentinel marker for severe systemic pathologies. **Aims:** This review aims at examining incidence, mechanisms, underlying causes, and clinical outcomes of SVT within the NVV population. **Materials and Methods:** We conducted a comprehensive narrative review of the existing medical literature. **Results:** SVT in NVVs is frequently associated with systemic conditions, including inherited or acquired thrombophilia, visceral or hematologic malignancies (notably Trousseau’s syndrome), vasculitis (e.g., Behçet’s syndrome), and connective tissue disorders. Specific manifestations like migratory SVT or Mondor’s disease provide crucial diagnostic clues. Notably, NVV-SVT carries a significantly higher risk of recurrence and venous thromboembolic events compared to VV-associated cases. **Conclusions:** A thorough diagnostic work-up is essential for patients with NVV-SVT to ensure early detection of underlying systemic diseases. Although current management does not differentiate between VV and NVV cases, the increased thromboembolic risk in the latter suggests a need for tailored therapeutic approaches. Further prospective studies are required to evaluate differentiated anticoagulant strategies regarding dosage and duration for this high-risk population.

## 1. Introduction

Superficial venous thrombosis (SVT) is an acute disease of the superficial venous circulation, characterized by thrombosis and an inflammatory response of the venous wall [1]. Clinically, SVT is diagnosed by detecting a red, painful, and palpable cord, followed by an assessment with an ultrasound scan to evaluate thrombus extension and rule out deep vein involvement [2]. Lower-limb veins are the most common site for SVT development, with varicose veins (VVs) being the main associated factor [3]. Varicose veins are abnormally dilated, tortuous (twisted), and elongated superficial veins in which the valves have become incompetent, leading to retrograde blood flow and increased venous pressure [4]. Consequently, SVT has historically been considered a benign condition due to its association with chronic venous disease (CVD) [5]. However, thrombosis can also occur in superficial non-varicose veins (NVVs) of the lower limb, including the saphenous veins and their tributaries. Although less frequent, SVT can also arise in NVVs of the upper extremities, groin, abdomen, chest, and penis [6,7].

This review aims to analyze the prevalence of SVT occurring in NVVs among all SVT cases, investigate the underlying causes, and examine trial data concerning its incidence and impact on patient outcomes.

## 2. Materials and Methods

A comprehensive search was conducted in November 2025 across major electronic databases, including PubMed and MEDLINE, using a combination of Medical Subject Headings (MeSH) and predefined keywords such as “ superficial venous thrombosis,” “venous thromboembolism”, “thrombosis”, “varicose vein”, “non-varicose vein”, “healthy vein”, “cancer associated thrombosis”, “gender vein thrombosis”, and “sex-related thrombosis”. No publication date restrictions were applied, and the selection was limited to English-language articles.

## 3. Epidemiology

Initial studies, based on clinical diagnoses of SVT, reported an annual incidence of 123,000 cases in the United States, along with a prevalence of 3% to 11% in the Swiss population. These studies, however, failed to provide specific data concerning SVT occurring in the absence of underlying venous disease [6].

Data from the one-year multicenter, community-based study conducted by the STEPH group in France [8], on an urban population, indicated that 171 adults received an SVT diagnosis, which was confirmed via ultrasound by vascular specialists. This finding translates to an annual diagnosis rate of 0.64% (95% CI, 0.55–0.74%). Annual rates varied significantly by age and sex, ranging from 0.04% (95% CI, 0.00–0.10%) in men aged 18–39 years to 2.19% (95% CI, 1.59–2.78%) in women aged ≥75 years. However, the authors cautioned that using a primary care setting may have resulted in an underestimation of the true prevalence. Furthermore, of the 171 diagnosed cases, 130 (76%) involved VVs, while 41 (24%) occurred in NVVs. At the time of SVT diagnosis, a significant proportion of patients had concomitant deep vein thrombosis (DVT) (24.6%) or pulmonary embolism (PE) (4.7%). The authors do not specify the proportion of SVT cases with concomitant DVT/PE that occurred in VVs versus NVVs [8].

Similarly, a retrospective cohort study utilizing coded data from the Utrecht General Practitioner Network database assessed the annual incidence of SVT in the Dutch population [9]. SVT diagnosis was clinically determined, relying on the reported description of typical signs and symptoms. The incidence of SVT events in the primary care setting was 1.31 per 1000 person-years of follow-up [9]. Notably, SVT involved a NVV in 60.5% of the total SVT population and in 83.5% of cases presenting with co-existing venous thromboembolism (VTE). The study concluded that the VTE risk was approximately 4.1%, primarily within the first month following SVT diagnosis, and was particularly elevated in patients with cancer and in those with SVT occurring in the absence of underlying varicose veins [9].

Another French epidemiological study, the POST study, assessed 844 patients with SVT and reported that 20% of the cohort presented with thrombosis in NVVs. Significantly, the percentage of NVVs was substantially higher in patients with concomitant DVT or PE (31.6%) compared to those with isolated SVT (13.7%). Crucially, the absence of VVs was identified as an independent risk factor for symptomatic VTE at 3 months, including recurrence or extension of SVT [Hazard Ratio (HR), 2.06; 95% Confidence Interval (CI), 1.01–4.25; *p* = 0.049] [10].

## 4. Anatomy, Physiology and Pathophysiology of Lower-Limb Venous Circulation

**Figure 1:**
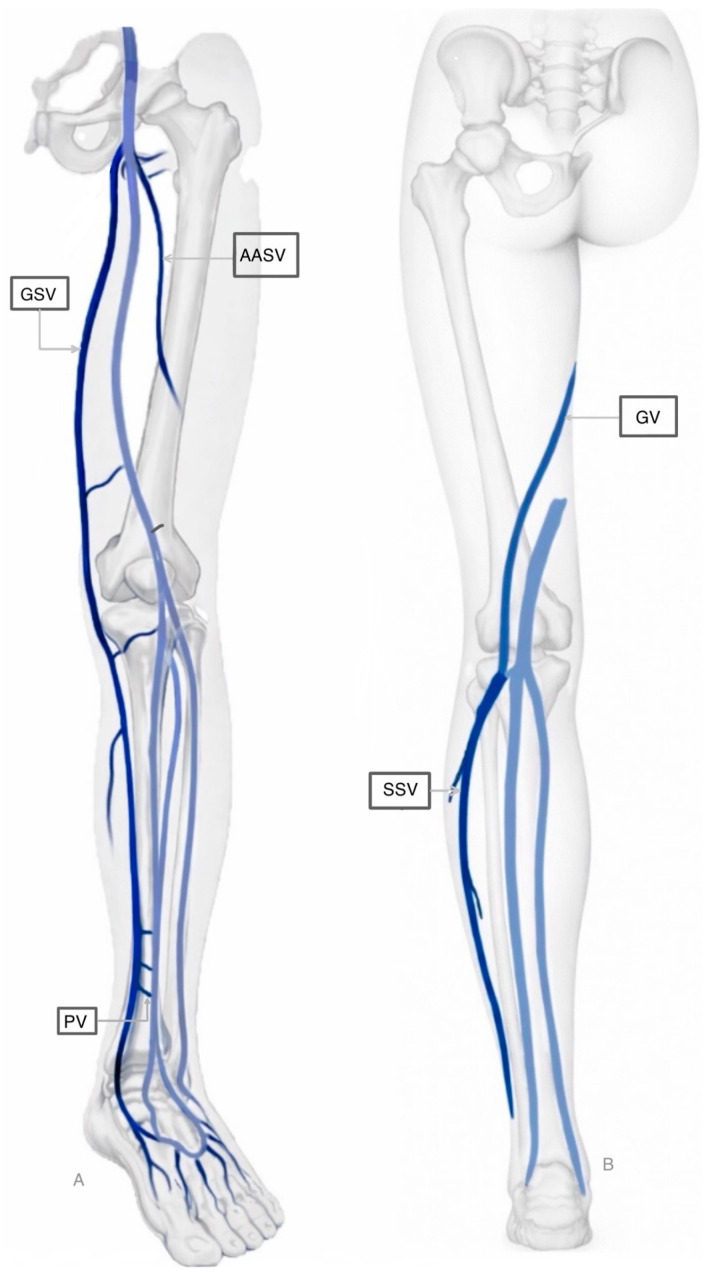
Superficial circulation of the lower extremities. Part A (Left): Antero-lateral view of the limb, primarily highlighting the Great Saphenous Vein and its connections. Part B (Right): Posterior view, showing the Small Saphenous Vein and the Giacomini Vein ascending toward the thigh. Deep veins are represented in light blue. Legend: GSV: Great Saphenous Vein; AASV: Anterior Accessory Saphenous Vein; SSV: Small Saphenous Vein; PV: Perforating Vein; GV: Giacomini Vein.

The venous circulation, which serves as the body’s blood reservoir by accommodating an estimated 60% to 75% of the total blood volume, is structured into three primary systems: the superficial, deep, and perforating veins [11]. These systems are classified according to their relationship with the muscular fascia of the lower limb. Superficial veins are situated epifascially (above the fascia) and drain the cutaneous microcirculation. In contrast, deep veins are located subfascially (beneath the fascia). Perforating veins traverse the fascia, effectively connecting the superficial and deep systems and thereby directing blood flow from the periphery into the deep venous network [12]. Figure 1 illustrates the superficial circulation of the lower extremities.

Venous blood from the lower extremities is propelled against gravity primarily by the action of the calf muscle pump and the foot venous pump. The prevention of reflux (backflow) is ensured by a network of one-way bicuspid valves distributed throughout the superficial, deep, and perforating venous systems. When an individual assumes an upright position, the lower limbs can pool approximately 500 mL of blood [13].

Pathological venous reflux arises when these physiological mechanisms are compromised, resulting in venous valve incompetence, vessel dilation, venous hypertension, and local inflammation.

Thrombus formation in SVT is closely linked to an inflammatory response of the venous wall. Its etiology is multifactorial, typically triggered by mechanical, chemical, biological, or infectious processes, as well as trauma. These factors involve the classic elements of Virchow’s triad: venous stasis, endothelial injury, and hypercoagulability [6]. While these factors often coexist, their relative contribution varies significantly between VVs and NVVs. In the context of VVs, the predominant mechanism is blood stasis coupled with chronic structural changes in the vessel wall [14]. the local accumulation of procoagulant factors, leukocytes, and platelets, all of which play a critical role in the initiation and propagation of the thrombus [15]. Furthermore, stasis alters endothelial function by downregulating protective antithrombotic factors and increasing the expression of adhesion molecules, such as P-selectin and von Willebrand factor (vWF).

P-selectin binds to P-selectin glycoprotein ligand-1 on leukocytes while vWF binds to specific receptors on platelets, leading to their activation. Once recruited, leukocytes express tissue factor (TF), further promoting the coagulation cascade [16].

Conversely, the pathophysiology of SVT in NVVs often shifts from a mechanical alteration to a biochemical and inflammatory phenomenon. In these cases, endothelial activation, and its subsequent shift toward a pro-inflammatory and hypercoagulable state, acts as the primary trigger [17]. Consequently, the coagulation cascade may be initiated even in the absence of significant stasis. In systemic conditions such as malignancy or autoimmune disorders, circulating pro-inflammatory cytokines and microparticles can induce a systemic hypercoagulable state [18,19]. These systemic triggers overcome the natural anticoagulant properties of the healthy endothelial lining, leading to ‘spontaneous’ thrombosis in vessels otherwise devoid of pre-existing reflux.

## 5. Sex- and Gender-Related Differences in SVT

The relationship between gender and SVT shows distinct patterns throughout life, with important interactions with sex hormones, age, and comorbidities. Varicose veins, the most important risk factor for SVT, are more prevalent in women than men [20]. Also, hormone changes during pregnancy and puerperium expose women to a higher risk of SVT [21].

The female predisposition to chronic venous insufficiency (CVI) is rooted in a complex interplay of hormonal and mechanical factors. Estrogen and progesterone receptors are expressed directly in the venous walls and valves and are increased in VVs [22].

Progesterone, in particular, promotes venous distensibility by reducing the tone of smooth muscle fibers in the vessel wall, leading to passive dilation and valvular incompetence. During pregnancy, higher serum progesterone levels are associated with greater prevalence of varicose veins/CVI; women with varicose veins had higher progesterone than pregnant controls [23].

In postmenopausal women, the decline in estrogen levels can lead to a loss of venous wall elasticity, which, when combined with age-related sedentary behavior, further exacerbates venous stasis [24]. However, the risk remains high if postmenopausal hormone therapy is administered, as exogenous hormones can restart the pro-thrombotic and vasodilatory pathways mentioned above [25].

A multicentre, cross-sectional, observational study that was conducted among 1536 patients attending general practitioner clinics who underwent ambulatory screening procedures showed that SVT was more prevalent in women than in men (2.5% vs. 1.5%). This increased prevalence was particularly notable in the presence of varicose veins and in patients aged over 50 years (or ≥50 years) [26].

Women with a history of SVT are at a substantially higher risk of VTE complications when acquired thrombotic risk factors are present. Data from the MEGA study [27]. showed that in women with a history of SVT, the presence of a reproductive risk factor (such as oral contraception, postmenopausal hormone therapy, or pregnancy and the puerperium) markedly increased the risk of VTE (see Section 6.2).

Conversely, regarding the risk of VTE complication (at three months following isolated SVT), the male gender emerges as a significant risk factor [Odds Ratio (OR), 2.17; 95% CI, 1.28–3.68; *p* = 0.004]. This risk is particularly elevated when the male sex is associated with severe CVI [28].

## 6. Predisposing Factors for SVT in NVVs

SVT development in NVVs is predisposed by a wide array of factors, including conditions promoting hypercoagulability (such as advanced age, cancer, inherited thrombophilias, and use of hormonal therapies), circumstances leading to venous stasis (e.g., prolonged immobility, pregnancy, and post-surgical states), and situations causing endothelial injury (including trauma, insertion of vascular devices, sclerotherapy, and infusion of hypertonic or vaso-damaging substances) [29,30,31] (Table 1).

### 6.1. Hereditary Thrombophilia

Samlaska and James introduced a distinction between primary and secondary hypercoagulable states in the context of SVT [32,33]. Clinical indicators suggestive of a primary hypercoagulable state include a family history of thrombosis, recurrent thrombotic events without identifiable precipitating factors, thrombosis in unusual anatomical sites, early onset of thrombotic episodes, and resistance to conventional antithrombotic therapy [32,33].

A study prospectively monitoring VTE events in patients with prior unprovoked VTE following the discontinuation of anticoagulant therapy identified SVT as a significant risk factor for VTE relapse. Furthermore, this research demonstrated that elevated Factor VIII levels constituted an independent risk factor for SVT itself [34].

The initial data regarding a potential correlation between SVT and protein S deficiency originated from a small study of 36 patients with recurrent SVT. This study reported that 5.5% of the cohort exhibited a protein S deficit. While the study was inherently limited by its small sample size and reliance on clinical diagnosis of SVT (lacking objective confirmation), the findings warranted further investigation [35].

Furthermore, another small-scale study suggested a correlation between the presence of antiphospholipid antibodies and recurrent SVT [36].

Karathanos and colleagues [37] conducted an investigation specifically examining the risk factors for SVT in patients with pre-existing VVs. Their study, which included 128 patients confirmed by ultrasonographic compression to have both conditions, identified significant associations between SVT and several factors. These included older age, male sex, obesity, and thrombophilia abnormalities, such as protein S deficiency. Notably, these associations were particularly pronounced in individuals with VVs of moderate disease severity [37].

Evidence suggests that individuals with SVT exhibit a two- to three-fold-higher prevalence of thrombophilia factors when compared to those without the condition. The frequent occurrence of hereditary thrombophilia—including factor V Leiden, the prothrombin mutation G20210A, or deficiencies in protein C, protein S, or antithrombin (AT)—indicates a pathogenesis that resembles DVT [38,39,40]. In support of this analogy, one study focusing on SVT in NVVs (albeit involving only 73 patients) revealed a thrombophilia prevalence of 76%. Within this cohort, 51% exhibited the Factor V Leiden mutation, with approximately half of those cases presenting as homozygous. The authors also reported significant associations with additional thrombophilia factors [41].

Conversely, an Italian retrospective study involving approximately 2000 patients presented conflicting data. Specifically, SVT was not associated with the Prothrombin G20210A mutation, antiphospholipid antibody positivity, hyperhomocysteinemia, or elevated Factor VIII levels. In contrast, the study reported a significant association between SVT and deficiencies in protein C, protein S, and AT III [42]. Furthermore, Milio et al. [43] showed an increased prevalence of the factor V mutation, particularly in cases of SVT occurring in NVVs and those extending into the deep venous system. Collectively, mutations such as Factor V Leiden, Prothrombin G20210A, and deficiencies in Protein C, Protein S, and AT III have been established as key risk factors for both the development of SVT and its progression to DVT, particularly when the SVT arises in the absence of VVs [44].

A small-scale study by Sobreira et al. [45], which investigated thrombophilia prevalence in SVT, reported that 74.2% of cases involved NVVs. Screening for thrombophilia was performed in 87.7% of the overall cohort, with 53.4% yielding positive results. Notably, a surprisingly high prevalence of positive screening (27.6%) was also documented in patients with VVs (25.8% of the cohort), where screening had been conducted in 80.9% of cases. The study identified deficiencies in Protein C (2.1%), Protein S (6.2%), and AT III (6.25%), alongside frequent detection of the Factor V (46.4%) and Prothrombin II G20210A (21.4%) mutations, both exclusively found in the heterozygous state [45].

Collectively, the aforementioned studies are often limited by methodological constraints, particularly small sample sizes [34,35,36,37,38,39,40,41,42,43,44,45]. Nevertheless, the consistent association demonstrated between SVT and genetic or acquired thrombophilia, especially in the context of NVVs SVT, warrants rigorous further investigation.

Current Brazilian guidelines and a consensus statement from the International Union of Angiology on SVT recommend thrombophilia screening under specific conditions [14,46]. These indications include SVT involving NVVs (after excluding occult malignancy), thrombosis progressing despite adequate treatment, recurrent SVT, and suspected autoimmune disease. While thrombophilia screening in VTE patients is debated, it is generally restricted to younger individuals and does not typically influence the choice or duration of anticoagulant therapy [47].

### 6.2. Pregnancy, Postpartum Period, Oral Contraceptives and Hormone Replacement Therapy

A Danish cohort study estimated the prevalence of SVT during pregnancy at approximately 0.1%. The authors also investigated the timing of SVT onset from conception through three months postpartum. The data indicated that SVT manifested predominantly during the postpartum period, recording an incidence rate of 1.6 per 1000 person-years (95% CI, 1.4–1.7). Throughout pregnancy, a gradual escalation in incidence was observed across trimesters: 0.1 (95% CI, 0.1–0.2) in the first, 0.2 (95% CI, 0.2–0.3) in the second, and 0.5 (95% CI, 0.5–0.6) in the third. A crucial finding was that 10.4% of women with a prior history of SVT subsequently developed VTE, underscoring the clinical significance of SVT within the maternal patient population [48].

A retrospective study involving 65 women who presented with venous thrombosis while receiving oral contraceptives and hormonal therapy reported that 5 of these cases were SVT. The findings also indicated that these women frequently presented with genetic thrombophilia and a family history of VTE. This suggests that the use of these therapies may confer an additive risk of both SVT and DVT/PE, particularly in individuals with a known familial predisposition to VTE [49].

Another study investigated the risk of VTE in patients with previous SVT and acquired risk factors. The previous SVT alone was associated with a 5.5-fold increased risk [adjusted OR (aOR), 5.5; 95% CI, 4.4–6.8]. In women, the combination of prior SVT and reproductive factors (oral contraception, postmenopausal hormone therapy, or pregnancy/puerperium) elevated the risk substantially to an aOR of 34.9 (95% CI, 19.1–63.8). The risk associated specifically with oral contraception was even higher, yielding an aOR of 43.0 (95% CI, 15.5–119.3) [27].

The Balkan Working Group for the Prevention and Treatment of Venous Thromboembolism developed a position paper outlining the management of SVT during pregnancy. The paper recommends that SVT management be continued until the end of gestation and extend through six weeks postpartum. Furthermore, the guidelines advise performing periodic ultrasound re-evaluation to actively exclude the presence of concomitant or progressive DVT [21].

### 6.3. Cancer

The initial documented association between thrombosis and cancer is historically attributed to the French physician Jean-Baptiste Bouillaud, who published a relevant manuscript in 1823 [50]. However, Armand Trousseau first provided a comprehensive clinical understanding of this mechanism in 1865, definitively describing the association between occult malignancy and venous thromboembolism, particularly recognizing migratory superficial vein thrombosis [51]. Trousseau astutely identified that thrombosis could arise from an underlying pathological blood condition, long predating a formal understanding of hypercoagulability [52]. His personal experience, succumbing to carcinoma two years after being diagnosed with phlebitis, cemented his legacy and popularized his eponymous syndrome (Trousseau Syndrome) [53].

The definition of Trousseau’s syndrome has undergone continuous refinement. Initially characterized by Sack et al. with chronic disseminated intravascular coagulopathy, microthrombi, and verrucous endocarditis [54], the diagnostic scope has since broadened to encompass virtually all cases of cancer-associated thrombosis, despite the heterogeneity of underlying mechanisms [18].

Trousseau’s syndrome results from a complex interplay between cancer pathophysiology and host hemostatic mechanisms, sustained by Virchow’s triad: blood stasis, endothelial injury, and a hypercoagulable state. Hypercoagulability, often the most significant factor, is linked to malignant cells’ production of procoagulant substances. A key mechanism involves the tumor cell overexpression of TF, the primary initiator of the extrinsic coagulation pathway. Elevated TF levels are frequently observed in specific malignancies (pancreatic, lung, brain tumors) and are potentiated by secreted TF-containing microparticles [55]. Additionally, many tumors produce mucins that enhance cellular adhesion by binding to selectins on platelets and endothelial cells. This prothrombotic environment is intensified by systemic inflammation, creating a self-reinforcing loop among inflammation, coagulation, and tumor progression [56]. Furthermore, the intrinsic coagulation pathway plays a pivotal role, with recent studies highlighting the contribution of Neutrophil Extracellular Traps (NETs). These DNA- and protein-based structures provide a crucial scaffold for clot formation and activate Factor XII, establishing a direct molecular link between inflammation and coagulation [57]. Notably, in myeloproliferative neoplasms such as essential thrombocythemia and polycythemia vera, oncogenic mutations—specifically the JAK2 V617F mutation—are known to drive heightened platelet activation and inflammation. This process predisposes patients to both arterial and venous thrombosis [55].

In conclusion, Trousseau’s syndrome is a multifactorial condition arising from a complex interplay between malignant cells, the vascular system, the immune response, and the coagulation cascade. A comprehensive understanding of these interconnected mechanisms is therefore essential to improve the diagnosis and management of affected patients. In this context, Ikushima et al. proposed a useful framework by classifying the risk factors for cancer-associated thrombosis into patient-related, treatment-related, and cancer-related categories [58].

Extensive research has focused on determining the prevalence of occult malignancies at SVT presentation and quantifying the risk of subsequent malignancy, aiming to establish clinical pathways for early cancer detection. A retrospective study reported a cancer prevalence of 8.7% in patients with SVT. Furthermore, the authors identified significant associations between age, thrombophilia, male sex, non-varicose SVT and the concurrent diagnosis of cancer, DVT, or PE. Notably, malignancy emerged as the most important risk factor for the development of DVT/PE within this patient cohort [59].

In a case–control study comprising 277 SVT cases and 553 controls, patients with a prior malignancy were excluded. The remaining cohorts—SVT (*n* = 250) and controls (*n* = 504)—were followed for 24 months, achieving adherence rates of 88% and 85%, respectively. Among participants cancer-free at baseline, new malignancies were diagnosed in 2% of the SVT group (5/250) and 2% of the control group (10/504). Mortality among patients with incident cancer was identical between groups, with two deaths recorded in each; all other cancer patients continued follow-up. Compared to the general Dutch population, no statistically significant increase in cancer incidence was observed within two years following an unprovoked SVT episode (Standardized Morbidity Ratio, 1.1; 95% CI, 0.5–2.7) [60].

In a prospective observational study of 632 patients referred for VTE, 205 presented with SVT, of whom 16.6% had a diagnosis of active cancer. Significant associations were identified between active malignancy and specific SVT subsets: proximal SVT [Risk Ratio (RR), 1.54; 95% CI, 1.18–2.03; *p* < 0.01], SVT within 3 cm of a junction (RR, 2.01; 95% CI, 1.13–3.72; *p* = 0.019), bilateral SVT (RR, 8.38; 95% CI, 2.10–33.43; *p* < 0.01), and multi-vein involvement (RR, 2.42; 95% CI, 1.40–4.20; *p* < 0.01). Furthermore, cancer was associated with a higher risk of both SVT persistence (RR, 1.51; 95% CI, 1.18–1.95; *p* < 0.01) and progression (RR, 5.75; 95% CI, 2.23–14.79; *p* < 0.01). Notably, patients without prior malignancy exhibited an increased risk of occult cancer detection during follow-up (RR 1.43; 95% CI, 1.13–1.18; *p* = 0.022), particularly in cases characterized by proximal or bilateral SVT, initial thrombus progression, or subsequent DVT/PE [61].

The OPTIMEV study [62], a French multicenter observational cohort, investigated the long-term prognosis of cancer patients presenting with isolated lower-limb SVT. Among 8256 consecutive patients enrolled for suspected symptomatic VTE, 599 were diagnosed with SVT, 34 of whom had cancer-related SVT. The authors compared the outcomes of these 34 oncology patients against three distinct comparator cohorts: 102 age- and sex-matched cancer-free patients with SVT, 68 cancer patients with DVT, and 27 cancer patients presenting with both SVT and concomitant DVT. Over a follow-up period extending up to three years, results demonstrated that isolated SVT in cancer patients is associated with a poor prognosis, yielding a mortality rate of 23.2% per patient-year (p-y). This rate was statistically comparable to the mortality observed in cancer patients with DVT [27.2% p-y; adjusted Hazard Ratio (aHR), 1.0; 95% CI, 0.6–1.9] and significantly higher than that of SVT patients without cancer (2.0% p-y; aHR, 0.1; 95% CI, 0.04–0.29; *p* < 0.05). Notably, patients with both cancer and concomitant SVT/DVT exhibited the highest mortality rate at 57.4% p-y (aHR, 2.2; 95% CI, 1.1–4.3; *p* < 0.05), suggesting that the presence of both conditions may serve as a marker for a particularly aggressive disease course.

Regarding thrombotic complications, cancer patients with SVT experienced DVT or PE at a rate of 6.0% p-y, which is similar to the 7.5% p-y observed in cancer patients with DVT (adjusted cause-specific hazard ratio [aCHR], 1.5; 95% CI, 0.4–5.8) and substantially higher than the 1.4% p-y recorded in non-cancer SVT patients (aCHR 0.3; 95% CI, 0.08–1.4). Furthermore, all recurrences within the cancer groups manifested as serious events (DVT or PE), whereas non-cancer patients tended to experience less severe recurrences of SVT. The risk of major bleeding among cancer patients with SVT was 2.9% p-y, a rate comparable to those with DVT (3.3% p-y; aCHR 1.1; 95% CI, 0.2–5.8) and notably higher than in the non-cancer SVT cohort (1.4% p-y; aCHR 0.7; 95% CI, 0.1–4.0).

Of the 16 cancer patients with SVT who died during follow-up, most deaths (81.3%) were attributed to cancer progression, with no fatalities resulting from PE and only one death due to bleeding. An analysis of clinical factors revealed that oncology patients whose SVT developed in VVs showed a lower, though not statistically significant, risk of death compared to those without VVs (aHR 0.6; 95% CI, 0.3–1.0). Additionally, while the presence of VVs was associated with more localized cancer in patients with DVT (66.7% vs. 47.5%), this correlation was not observed in the SVT group. Finally, given that approximately 44.3% of oncology patients with SVT also presented with concomitant DVT, these findings underscore the necessity of systematic duplex ultrasonography to rule out DVT in any oncology patient presenting with SVT [62].

Complementing these findings, the IROVAM-ISVT systematic review and meta-analysis evaluated the incidence and risk of VTE in patients with isolated SVT and active malignancy [63]. This investigation was prompted by the clinical complexities of managing cancer-associated SVT, a condition characterized by a narrow therapeutic window due to the concurrent high risks of recurrent VTE and major bleeding. The meta-analysis synthesized data from eight full-text studies, encompassing a total of 5998 patients with SVT, of whom 448 (7.47%) were identified as having an active malignancy. The primary analysis revealed a VTE incidence rate of 18.2 events per 100 patient-years (95% CI, 5.2–31.2) among patients with cancer-associated SVT. This cohort exhibited a significantly elevated VTE risk compared to individuals without active malignancy (RR, 2.57; 95% CI, 1.78–3.71; *p* < 0.001). Concurrently, the rate of major bleeding was recorded at 2.0 events per 100 patient-years. Notably, the risk of VTE-related hospitalization was markedly higher in the active malignancy group (HR, 8.87; 95% CI, 2.12–37.12; *p* = 0.003).

The systematic review further delineated specific incidence rates for secondary outcomes within the cancer-associated SVT population: PE at 9.6 events per 100 patient-years, DVT at 20.1 events per 100 patient-years, and all-cause mortality at 22.8 events per 100 patient-years for patients with cancer-associated SVT. In conclusion, patients with isolated SVT and active malignancy experience substantial rates of VTE despite current therapeutic interventions. These findings highlight a critical unmet need and underscore the necessity for prospective trials to evaluate the efficacy and safety of extended anticoagulation durations in this high-risk population [63].

### 6.4. Autoimmune Diseases

Autoimmune disorders have been established as significant factors in the pathogenesis of SVT. In Behçet’s disease, vascular involvement is attributed to its characteristic vasculitis that affects both arterial and venous vessels of all calibers [64].

Distinctively, a unique transmural inflammation involving all three vessel layers has been documented in these patients [65].

Vascular manifestations are prevalent, occurring in up to 40% of cases, and frequently precede the fulfillment of formal diagnostic criteria [66]. The pathophysiology of Behçet’s syndrome involves complex thromboinflammatory mechanisms, where neutrophils and NETs play a pivotal role in sustaining chronic inflammation and vascular endothelial damage. These mechanisms necessitate a multimodal therapeutic approach, typically combining immunosuppressive agents with anticoagulants [19,67,68]. Similarly, patients with thromboangiitis obliterans, also known as Buerger’s disease, are highly susceptible to SVT. This rare inflammatory condition targets small and medium-sized arteries and veins, primarily affecting male heavy smokers. SVT is observed in 40% of these cases, often presenting with a migratory pattern that may serve as a clinical precursor to ischemic arterial symptoms in the affected extremities [69,70].

Finally, Mondor’s disease represents a specialized clinical entity of SVT, localized to the veins of the anterolateral chest wall, penis, or axillary region—the latter often following axillary surgery [71].

Although mechanical triggers such as trauma, surgery, or strenuous physical exertion are well-recognized, underlying prothrombotic states and systemic inflammatory conditions are increasingly implicated. Specifically, recurrent or atypical presentations of Mondor’s disease have been associated with protein S deficiency, autoimmune disorders, and occult malignancies [72].

### 6.5. Peripheral Venous Catheters: Endothelial Damage and the Progression to Suppurative SVT

In the upper extremities and neck, SVT is predominantly iatrogenic, arising most frequently from the insertion of intravenous catheters for chemotherapy, parenteral nutrition, or pharmacological infusions. It represents a common complication among hospitalized patients, typically localizing in the veins of the forearm or hand [73].

A wide range of intravenous medications and solutions are recognized for their potential to induce direct vascular endothelial injury. Agents such as diazepam, pentobarbital, calcium gluconate, and concentrated potassium chloride—along with specific antibiotics (e.g., vancomycin, nafcillin), chemotherapeutic protocols, and total parenteral nutrition mixtures—are well-documented endothelial toxins associated with vascular complications [73]. Factors predisposing to chemical thrombophlebitis include the selection of distal infusion sites, extended catheter dwell time, and hyperosmolar solutions (>800–900 mOsm/L). The underlying pathophysiology involves acute endothelial dysfunction, the triggering of pro-inflammatory cascades, and subsequent mural thrombus formation [73,74].

Suppurative SVT is a rare yet severe infectious sequela of superficial venous thrombosis, characterized by the purulent infection of the thrombus within an inflamed vein. The etiology is frequently associated with compromised skin integrity, peripheral intravenous catheterization, or contaminated injection sites (particularly in the context of intravenous drug use). The predominant pathogens implicated include Staphylococcus aureus, notably methicillin-resistant strains (MRSA), Streptococcus pyogenes, and various Gram-negative bacilli [33]. Clinical presentation typically includes localized pain, erythema, and pyrexia, often accompanied by purulent drainage at the site of injury and, in some cases, systemic inflammatory response syndrome or sepsis. Diagnosis is established through clinical examination, confirmatory blood cultures, and imaging—most notably color Doppler ultrasonography. Management necessitates targeted systemic antibiotic therapy and, frequently, surgical excision of the infected venous segment. Prompt recognition is imperative to mitigate the risk of life-threatening complications, including septic embolization and proximal thrombus extension [75].

## 7. Treatment of SVT in NVVs: Results from Clinical Trials

The STENOX TRIAL [76] evaluated the efficacy of a 12-day therapeutic regimen for SVT. Participants were randomized into four treatment arms: oral placebo, oral tenoxicam, daily enoxaparin (40 mg), and weight-adjusted enoxaparin (1.5 mg/kg). Patients were stratified according to the severity of CVI ranging from asymptomatic to severe. Although the presence of varicose veins was not explicitly recorded, only 10 of the 427 participants were classified as asymptomatic. By day 12, a significant reduction in the composite endpoint of DVT and SVT recurrence was observed in all active treatment cohorts compared to the placebo group [76].

The study conducted by Vesalio Investigators Group enrolled 164 patients with SVT of the Great Saphenous Vein (GSV). The trial compared two doses of nadroparin: a therapeutic dose for 10 days, followed by a 50% dose reduction for the subsequent 20 days, versus a continuous prophylactic-dose regimen. Both treatment protocols were maintained for a total duration of one month [77]. SVT in NVVs occurred in 29.6% (24/81) of patients receiving prophylactic-dose anticoagulation, compared to 33.7% (38/83) of those administered a therapeutic regimen. The primary endpoint was a composite of symptomatic SVT extension, symptomatic DVT, and fatal or non-fatal PE over a 3-month follow-up period. No statistically significant differences were detected between the two study arms. Notably, 71.4% of adverse events—primarily SVT progression—manifested within the first month of follow-up in the prophylactic-dose group. Consequently, the authors suggest that a one-month therapeutic-dose regimen may offer superior protection against SVT progression [77].

The STEFLUX [78] was a controlled trial that enrolled 664 patients with isolated lower-limb SVT exhibiting a minimum lesion length of 4 cm. Patients were excluded if the SVT was located within 3 cm of the saphenofemoral junction (SFJ) or saphenopopliteal junction (SPJ). Participants were randomized to one of three treatment arms: (A) intermediate dose of Low-Molecular-Weight Heparin (LMWH) (Parnaparin 8500 International Unit (IU) once daily) for 10 days, followed by a placebo for 20 days; (B) intermediate dose of LMWH for 30 days (Parnaparin 8500 IU once daily for 10 days followed by 6400 IU once daily for 20 days); or (C) prophylactic dose of LMWH (Parnaparin 4250 IU once daily) for 30 days.

NVV-SVT was reported in 166/664 (25%) of the total patients participating in the study without significant differences between samples.

The primary outcome, a composite of symptomatic and asymptomatic DVT, PE, and SVT recurrence, was significantly lower in the 30-day intermediate-dose group (1.8%) compared to the shorter intermediate-dose group (15.6%) and the prophylactic-dose group (7.3%). These findings from the STEFLUX study therefore suggest that a 30-day intermediate dose of LMWH is more effective than either a shorter duration or a prophylactic dose. However, authors found that the clinical benefit of intermediate-dose over prophylactic-dose treatment diminished after 3 months [78]. The post hoc analysis of the STEFLUX study revealed that several specific factors were associated with a higher risk of VTE development, particularly following treatment discontinuation. These risk factors included the absence of varicose veins (OR, 2.6; 95% CI, 1.3–5.0; *p* = 0.007) and a family history of VTE (OR, 2.6; 95% CI, 1.4–4.8; *p* < 0.001). These findings underscore the importance of identifying high-risk individuals who may benefit from tailored anticoagulation strategies regarding both treatment duration and dosage [79].

The CALISTO study, a randomized, double-blind trial, enrolled 3002 patients to evaluate the efficacy and safety of fondaparinux compared to placebo for the treatment of lower-limb SVT [80]. Of the total cohort, only 342 patients (11.4%) in both groups presented with SVT in NVVs, and the prevalence of cancer was low (2.1% in the fondaparinux group and 1.9% in the placebo group). Treatment with fondaparinux at 2.5 mg/day significantly reduced the primary endpoint compared to placebo. Regarding safety, major bleeding was rare, with adverse events reported in 0.7% of the fondaparinux group and 1.1% of the placebo group. The primary composite outcome (all-cause death, symptomatic VTE, thrombus extension to the SFJ, or SVT recurrence) at day 47 occurred in 0.9% of the fondaparinux group versus 5.9% in the placebo group, representing a statistically significant relative risk reduction of 85% (*p* < 0.001) [80]. These findings provided the evidence base for current SVT management guidelines [81,82].

The SURPRISE trial [83] randomized 472 with SVTs extending at least 5 cm, who also possessed at least one additional risk factor (age > 65 years, male sex, prior VTE, cancer, autoimmune disease, or NVV-SVT), to receive either rivaroxaban 10 mg or fondaparinux 2.5 mg. In this study, NVV-SVT was present in 66/236 (28%) patients in the rivaroxaban group and 76/236 (32%) in the fondaparinux group. Over a 45-day period, the results demonstrated that rivaroxaban (10 mg) was non-inferior to fondaparinux (2.5 mg) regarding the composite endpoint of DVT/PE, SVT progression/recurrence, and all-cause mortality. The absence of major bleeding in both groups suggests that rivaroxaban, a more cost-effective oral option, represents a viable alternative to fondaparinux for the management of high-risk SVT patients [83].

A prospective study conducted by Nikolakopoulos et al. [84] enrolled 147 patients with isolated SVT, defined as a thrombus length exceeding 5 cm and a distance of more than 3 cm from the SFJ. Notably, CVI was the underlying cause of SVT in all cases; consequently, no cases of SVT in NVVs were reported. Participants were allocated into two groups: Group A received tinzaparin at variable dosages for 60 days, while Group B received an intermediate dose of tinzaparin (75% of the therapeutic dose) for 90 days. The findings revealed that the recurrence of thromboembolic events was significantly higher in the shorter-duration group (*p* = 0.004). Despite acknowledged limitations—including the non-randomized design and the lack of standardized dosing—the results suggest that an extended three-month tinzaparin regimen may be a viable strategy for preventing DVT complications in specific patient subsets, particularly those with axial SVT or multi-segmental involvement [84].

In a pooled analysis of two prospective studies involving a cohort of 956 patients treated with an intermediate dose of tinzaparin (131 IU/kg for 30 days), 357 (37.3%) presented with NVV-SVT. The studies evaluated 3-month outcomes, including DVT, PE, thrombus extension to the SFJ/SPJ, and recurrent SVT. The results indicated that VTE recurrence was not significantly associated with the type of vein affected, but was uniquely linked to the baseline thrombus length [85].

The INSIGHTS-SVT study prospectively collected data on the 3-month outcomes of patients with isolated SVT, specifically focusing on symptomatic DVT, PE, and the progression or recurrence of SVT. In the study population, 283 of 1159 patients (24.4%) were NVV-SVT. Among the 1150-patient cohort eligible for analysis, the incidence of recurrent or extended SVT, DVT, and PE was 4.7%, 1.7%, and 0.8%, respectively. After adjusting for propensity score and treatment duration, fondaparinux demonstrated superior efficacy regarding the primary outcome compared to LMWH. Multivariate analysis identified previous SVT (HR, 2.3; 95% CI, 1.44–7.78; *p* = 0.001), age (HR, 0.97; 95% CI, 0.96–0.99; *p* = 0.008), treatment duration (HR, 0.92; 95% CI, 0.83–0.99; *p* = 0.046), and thrombus length (HR, 1.03; 95% CI, 1.02–1.05; *p* < 0.001) as significant predictors of the primary outcome. Notably, treatment adherence of at least four weeks was significantly higher with fondaparinux than with LMWH (70% vs. 28%). When therapy exceeded 38 days, fondaparinux was associated with a lower rate of VTE recurrence (3.7% vs. 10.5%; HR, 0.37; 95% CI, 1.03–7.03; *p* = 0.044) [86].

A 12-month follow-up analysis of the INSIGHT-SVT study included 872 of the 1159 original patients: 19.9% of these presented with SVT involving NVVs, confirming an increased risk of VTE. In the multivariate analysis, the occurrence of acute SVT on VVs was not significantly associated with the development of thromboembolic events between 3 and 12 months (HR, 1.74; 95% CI, 0.60–5.01; *p* = 0.31). The authors concluded that a prolonged therapeutic approach might be reasonable for patients with specific risk factors, such as high BMI and a history of VTE [87].

A further sub-analysis of the INSIGHTS-SVT registry demonstrated that patients with cancer remain at significant risk of thromboembolic complications, even while receiving antithrombotic therapy. Although most events occurred within the first 3 months (HR, 3.63; 95% CI, 1.79–7.35; *p* < 0.001), this risk remained elevated throughout the 1-year follow-up (HR, 2.40; 95% CI, 1.30–4.45; *p* = 0.005). There was no significant difference in the prevalence of SVT on VVs between patients with and without cancer (809/1074 [75.3%] vs. 62/77 [80.5%]; *p* = 0.305). These findings underscore the importance of recognizing the heightened VTE risk in oncological patients and suggest that a prolonged or intensified anticoagulation regimen should be considered on an individual basis [88]. The study data are summarized in Table 2.

## 8. Discussion

The present review highlights that SVT occurring in NVVs is not merely a localized inflammatory event, but a significant clinical marker for systemic hypercoagulability and adverse thromboembolic outcomes. While SVT in VVs is linked to CVD pathophysiology, such as inflammation and local hemodynamic changes, NVV-SVT appears to share a closer pathophysiological profile with DVT.

Epidemiological data consistently show that while VVs remain the primary site for SVT [8,9,10], a substantial and variable proportion (20% to 60%) of cases occur in NVVs. Crucially, the absence of VVs is not a sign of a milder disease; on the contrary, it serves as an independent “red flag” for VTE complications. In the OPTIMEV study SVT in NVVs was significantly more frequently associated with a concurrent DVT or a PE than SVT on varicose veins (39.4% vs. 23.3%; *p* < 0.001) [89]. Furthermore, as shown in the POST study, the risk of symptomatic VTE at 3 months doubles in patients without VVs (HR 2.06), including recurrence or extension of thrombosis [10].

The occurrence of SVT in a NVVs is a risk factor for recurrence and for the extension of the thrombosis to the deep venous system.

NVV-SVT may represent the initial clinical manifestation of underlying pathologies. In particular, its association with malignancy remains a critical clinical concern. Trousseau’s syndrome exemplifies the complex interplay between cancer-related TF expression, systemic inflammation, and thrombosis. The OPTIMEV and IROVAM-ISVT data [62,63] underscore that cancer patients with SVT face a mortality and VTE recurrence rate comparable to those with DVT. The finding that 44% of oncology patients with SVT also have concomitant DVT [62] reinforces the need for an aggressive diagnostic and therapeutic approach in this subpopulation.

The assessment of hypercoagulable states is particularly relevant in NVV-SVT. According to the data shown, a high prevalence of hereditary thrombophilia (up to 76% in some cohorts) is found in patients with NVV-SVT, with Factor V Leiden and Prothrombin G20210A mutations being the most frequent [6].

The clinical significance of SVT extends beyond local vascular inflammation, often serving as a diagnostic precursor for systemic autoimmune conditions. As described above, vascular manifestations in Behçet’s disease can precede formal diagnosis in up to 40% of cases [66]. Similarly, the migratory pattern of SVT in Buerger’s disease often acts as a precursor for subsequent ischemic arterial events [69,70]. These findings reinforce the role of SVT as a ‘sentinel sign’ that necessitates a systemic rather than purely localized diagnostic approach.

Another important point we have highlighted in this review is the gender paradox: while SVT is more prevalent in women due to VVs and reproductive factors (pregnancy, oral contraceptives) [27], men with isolated SVT appear to have a higher risk of VTE complications (OR, 2.17; 95% CI, 1.28–3.68; *p* = 0.004) [28]. This highlights the importance of incorporating gender-specific risk assessments into clinical decision-making.

Current guidelines recommend the use of fondaparinux 2.5 mg for a total of six weeks in the treatment of SVT [81,82], with no distinction between thrombosis in VVs or NVVs. This indication derived from the CALISTO trial [80], where only 11.4% of patients presented with SVT in NVVs.

Other evidence suggests that NVV-SVT may require more intensive or prolonged anticoagulation; indeed the post hoc analysis of the STEFLUX trial demonstrated that patients with SVT in NVVs have a 2.6-fold increased risk of developing VTE compared to those with varicose veins [79]. This finding is further supported by the INSIGHTS-SVT study, which confirmed that NVV-SVT is associated with an increased long-term risk of thromboembolic events, persisting up to 12 months [87]. Therefore, the Vesalio investigators suggested that a one-month Nadroparin therapeutic-dose regimen might offer superior protection against progression [77]; the STEFLUX trial clearly showed that an intermediate dose of LMWH (Parnaparin) for 30 days was more effective than shorter or prophylactic regimens [78]. However, the diminishing benefit observed after three months in the STEFLUX study [78], coupled with the findings from Nikolakopoulos et al. [76], suggests that in high-risk subsets—such as those with NVV-SVT—an extended three-month regimen might be more appropriate to prevent late recurrences. Notably, the European Society for Vascular Surgery (ESVS) guidelines on the management of venous thrombosis recommend that for patients with SVT of the leg exhibiting high-risk clinical or anatomical features, a three-month course of anticoagulation may be considered [90].

Extended reduced-dose direct oral anticoagulants (DOACs) regimens have demonstrated a favorable net clinical benefit both in patients with cancer-associated thrombosis after the initial six months and in those with provoked VTE who remain at a heightened risk of recurrence [91,92], as well as in real-world practice [93]. However, reduced-dose DOAC regimens for VTE secondary prevention must be strictly administered according to clinical guidelines, as suboptimal management may increase the risk of long-term sequelae, such as post-thrombotic syndrome and rare complications like venous aneurysms [94].

The SURPRISE trial provided crucial evidence for the use of direct oral anticoagulants, demonstrating that Rivaroxaban 10 mg is non-inferior to Fondaparinux in patients with at least one additional risk factor, including the presence of NVV-SVT [75].

The TROLL registry highlights the challenges of long-term SVT management, reporting a 15.9% cumulative 5-year VTE incidence despite pharmacological intervention. Of particular clinical concern is the 20% failure rate observed with the rivaroxaban 10 mg regimen, which required up-titration to maintain therapeutic efficacy [95].

In light of these data, which suggest failures driven by complex pathophysiological mechanisms, there is a compelling rationale to reconsider the use of LMWH in this high-risk subset, particularly considering its beneficial pleiotropic effects [96].

The accurate identification of high-risk SVT cohorts is of paramount economic relevance, as it facilitates targeted prevention strategies that mitigate the substantial costs associated with VTE-related morbidity [97].

**Figure 2:**
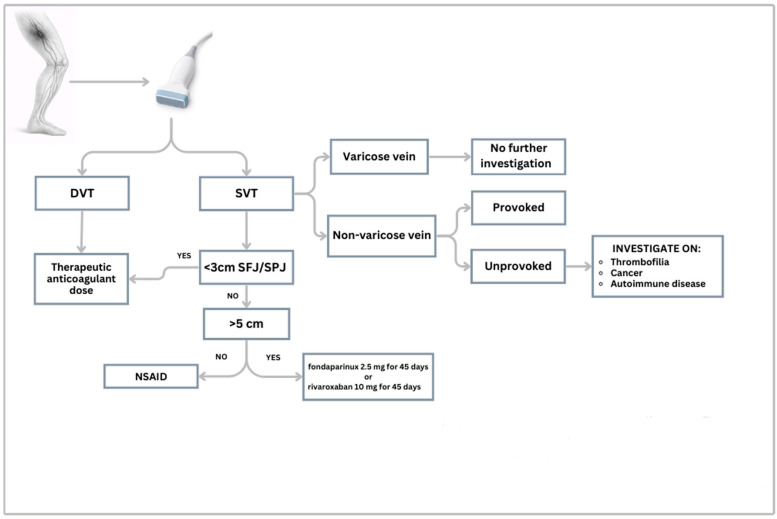
Diagnostic-therapeutic algorithm for suspected SVT. Legend: DVT: Deep Venous Thrombosis; SVT: Superficial Vein Thrombosis; SFJ: Sapheno-Femoral Junction; SPJ: Sapheno-Popliteal Junction; NSAID: Non-Steroidal Anti-Inflammatory Drugs.

A recent consensus from the French Society of Vascular Medicine distinguishes between provoked SVT, occurring in the presence of an identifiable and reversible risk factor, and unprovoked SVT, which occurs in the absence of any transient clinical triggers. This change in definition aims to significantly alter the approach to SVT by distinguishing the two forms with a different clinical, diagnostic, and therapeutic strategy [98]. Consequently, to ensure precision in clinical decision-making and optimize long-term outcomes, we propose a dedicated diagnostic and therapeutic algorithm, as delineated in Figure 2.

## 9. Conclusions, Limitations and Future Directions

SVT in NVVs is far from being a localized or benign vascular event as it could be a significant clinical indicator of systemic pathology, often preceding the diagnosis of occult malignancies, inherited thrombophilic states, or complex autoimmune disorders. Although most studies analyzed are limited by small sample sizes or retrospective designs, NVV-SVT is associated with a markedly higher incidence of recurrence and a greater propensity for progression toward deep vein thrombosis and pulmonary embolism. Consequently, a diagnosis of NVV-SVT must prompt clinicians to move beyond symptomatic treatment, initiating a targeted diagnostic work-up to exclude serious systemic triggers.

Current international guidelines do not yet provide differentiated therapeutic protocols for NVV-SVT, potentially leading to sub-optimal treatment in high-risk individuals.

Future prospective trials should specifically stratify patients by the presence or absence of VVs to determine if NVV-SVT requires a different therapeutic regimen rather than the standard SVT treatment.

Research on Artificial Intelligence (AI) for SVT is currently limited. However, AI tools used in broader venous imaging have already shown they can improve thrombus detection, reduce human error, and help create more consistent diagnostic workflows [99,100]. When incorporated into interpretable clinical decision-support algorithms, these AI tools may facilitate risk stratification and management decisions in SVT, particularly in NVVs where systemic risk factors and subtle imaging findings play a predominant role.

## Figures and Tables

**Table 1:** Risk factors for SVT in NVVs.

Risk Factors	
Obesity	Age > 75 years
Recent Surgery, Trauma	Active Cancer
Hypercoagulable states/Sepsis	Thrombophilia (Hereditary/Acquired)
Use of oral contraceptives, hormonal therapies, pregnancy	Inpatient Status, Immobilization
Previous DVT/PE	Mondor’s disease, Buerger’s disease
Infusion of endothelium-damaging substances	Autoimmune diseases
Male sex	Heart/respiratory failure

Legend: DVT: Deep Venous Thrombosis; PE: Pulmonary Embolism; NVV: Non varicose vein; SVT: Superficial Vein Thrombosis.

**Table 2:** Trial-Based Prevalence of Non-Varicose Vein Superficial Vein Thrombosis.

Trial	Treatment Strategy	Patients	SVT in NVVs	Key Outcomes
STENOX	Enoxaparin (prophylactic or therapeutic) vs. tenoxicam vs. placebo	427	2.1% asymptomatic CVI	- Enoxaparin significantly reduced combined SVT/DVT/PE - High-risk factors: male sex, prior VTE, severe CVI
VESALIO GROUP	Nadroparin (therapeutic + tapered dose) vs. prophylactic	164	- 29.6% NVV-SVT in prophylactic group - 33.7% NVV-SVT therapeutic group	- Higher event rate in prophylactic group - Fewer recurrences with therapeutic dose
STEFLUX	Parnaparin intermediate vs. prophylactic doses (10–30 days)	664	25% NVV-SVT	- 30-day intermediate dose had lowest events (1.8%) - Higher risk after treatment in NVVs
CALISTO	Fondaparinux 2.5 mg/day vs. placebo (45 days)	3002	11.4% NVV-SVT	85% RRR in thromboembolic events with fondaparinux, excellent safety
SURPRISE	Rivaroxaban 10 mg/day vs. fondaparinux 2.5 mg/day (45 days)	472	- 28% NVV-SVT in Rivaroxaban group - 32% NVV-SVT in Fondaparinux group	Non-inferior efficacy; no major bleeding in either group
Nikolakopoulos et al.	Tinzaparin 60 vs. 90 days (variable/intermediate dose)	147	0 NVV-SVT	90-day treatment reduced recurrence in high-risk SVT including multiple or NVVs
Tinzaparin Pooled Analysis	Tinzaparin 131 IU/kg/day for 30 days	956	37.3% NVV-SVT	- Recurrence predicted by thrombus length - Treatment duration not a significant factor
INSIGHTS-SVT	Fondaparinux ≥ 38 days vs. LMWH	1159	24.4% NVV-SVT	Fondaparinux group had fewer recurrent events (3.7% vs. 10.5%)

Legend: CVI: Chronic Venous Insufficiency; DVT: Deep Venous Thrombosis; IU: International Unit; LMWH: Low-Molecular-Weight Heparin; NVV: Non-Varicose Vein; PE: Pulmonary Embolism; RRR: Relative Risk Reduction; SVT: Superficial Vein Thrombosis.

## Data Availability

No new data were created or analyzed in this study. Data sharing is not applicable to this article.

## Abbreviations

The following abbreviations are used in this manuscript:

AI	Artificial Intelligence
AT	Antithrombin
CVD	Chronic venous disease
CVI	Chronic venous insufficiency
DOAC	Direct Oral Anticoagulant
DVT	Deep vein thrombosis
GSV	Great Saphenous Vein
LMWH	Low-Molecular-Weight Heparin
NETs	Neutrophil Extracellular Traps
NVV	Non-varicose vein
PE	Pulmonary Embolism
SFJ	Saphenofemoral junction
SPJ	Saphenopopliteal junction
SVT	Superficial Vein Thrombosis
TF	Tissue Factor
VTE	Venous Thromboembolism
VV	Varicose vein
vWF	von Willebrand factor
